# Osteogenesis of adipose-derived stem cells from patients with glucose metabolism disorders

**DOI:** 10.1186/s10020-020-00192-0

**Published:** 2020-07-02

**Authors:** Aleksandra Skubis-Sikora, Bartosz Sikora, Agnieszka Witkowska, Urszula Mazurek, Joanna Gola

**Affiliations:** 1 Department of Cytophysiology, Chair of Histology and Embryology, Faculty of Medical Sciences in Katowice, Medical University of Silesia in Katowice, ul. Medyków 18, C2/108, 40-752 Katowice, Poland; 2 Fundacja Zdrowego Życia, ul. Kotlarza 6, 40-139 Katowice, Poland; 3 Józef Tyszkiewicz Higher School in Bielsko-Biała, ul, Nadbrzeżna 12, 43-300 Bielsko-Biała, Poland; 4 Department of Molecular Biology, Chair of Molecular Biology, Faculty of Pharmaceutical Sciences in Sosnowiec, Medical University of Silesia in Katowice, Katowice, Poland

**Keywords:** Metformin, Mesenchymal stem cells, Metabolic impairment, type 2 diabetes, insulin resistance

## Abstract

**Background:**

Adipose derived stem cells (ADSCs) are clinically widely used somatic stem cells obtained from white adipose tissue. They are characterized by ability to differentiate e.g. into osteoblasts and might successfully regenerate bone tissue in fracture repair. However, the main problem of somatic stem cells is a documented influence of various diseases, drugs or age which can inhibit cells activity. Therefore, in the present study, we investigated the influence of insulin resistance (IR) and type 2 diabetes (T2D) on the proliferation and differentiation potential of ADSCs.

**Methods:**

The fat from subcutaneous abdominal adipose tissue was acquired by lipoaspiration from 23 voluntary participants, divided into three groups: with diabetes type 2, with insulin resistance and control healthy donors. The proliferative potential was analyzed by cell cytotoxicity assays and by mRNA expression of genes connected with proliferation. Flow cytometry was done for identifying proteins characteristic for mesenchymal stem cells and an analysis of osteogenic differentiation potential based on the assessment of osteogenic markers by real time RT-qPCR, and the evaluation of calcium deposition were also performed.

**Results:**

The results showed that diabetes type 2 lowered the activity of ADSCs in proliferation assays and changed their phenotypical characteristics. Interestingly, we observed differences in the proliferation potential of ADSCs in patients with insulin resistance, which is often the first phase of diabetes, compared to the control. It might suggest that insulin resistance, early-stage T2D, alters the activity of cells. Moreover, expression of osteogenesis markers was higher in cells from T2D patients than in cells from patients with IR and control.

**Conclusion:**

We conclude that type 2 diabetes changes the activity of stem cells, and insulin resistance influences on the proliferation of ADSCs.

## Introduction

Diabetes mellitus is a chronic metabolic disease that is caused by abnormalities in insulin secretion and by disorders in the hormone signaling pathway (Jiao et al. 2015). Type 1 diabetes (T1D), called insulin-dependent diabetes, is characterized by the loss of the ability of beta cells to produce insulin. The basis for this phenomenon is an autoimmune process that destroys beta cells either by apoptosis or necrosis (Stankov et al. 2013). Type 2 diabetes (T2D), known as non-insulin-dependent diabetes, accounts for 90–95% of all diabetes cases, and its main cause is insulin resistance (IR) (Simpson et al. 2015). If functioning properly, beta cells secrete an optimum amount of insulin, but this amount is not sufficient to compensate for the resistance of tissues to the hormone. Factors predisposing for this condition are obesity, physical inactivity, and age. T2D usually occurs in adults, often in the elderly (Simpson et al. 2015). Effective therapy for this type of diabetes is physical activity (O’Hagan et al. 2013), a proper diet (Evert et al. 2013), and pharmacotherapy (Amin and Suksomboon 2014).

The impact of diabetes on bones formation is very complex. Osteogenesis is adversely affected by abnormalities in the insulin signaling pathway (Evert et al. 2013). Activation of insulin receptors in osteoblasts stimulates their proliferation and induces the synthesis of collagen and osteocalcin. Osteocalcin in turn stimulates beta-cell proliferation and the secretion of insulin as well as increases testosterone production by the Leydig cells. The abnormal activity of insulin therefore leads to a reduction in osteocalcin production, which exacerbates insulin deficiency and leads to decreased testosterone production. Both osteocalcin and testosterone are osteogenic factors, and their reduced production negatively affects bone mass and the process of bone remodeling, which increases the risk of fractures (Yan and Li 2013).

Mesenchymal stem cells (MSCs) are somatic, multipotent cells that can be derived from different tissues. One of the richest sources of MSCs is adipose tissue (adipose-derived stem cells [ADSCs]). There are many experiments that show the advantages of using MSCs in regenerative medicine (Dzhoyashvili et al. 2014), though some studies suggest that abnormal insulin activity negatively affects MSCs, causing the loss of their proliferative potential and weakening their differentiation potential into osteoblasts (Yan and Li 2013). However, most of the research focus on experiments with animal models or on standardized cells from cell banks in vitro. These experiments only create a condition similar to human organism and fully studying complex interaction is not possible.

Therefore, the aim of the study was to analyze the activity of ADSCs from patients who did not receive any treatment and had insulin resistance, which is a precursor stage to type 2 diabetes, and also cells from patients with T2D in comparison to non-diabetic patients.

## Material and methods

### Patients

The fat from subcutaneous abdominal adipose tissue was acquired by lipoaspiration from 23 participants. All participants were divided into three groups: T2D group with 9 patients (3 men, 6 women, average age 45,3 years, average time of diabetes 6,2 years, all of them took metformin in highest tolerable doses, average dose 1,5 g/d), IR group with 6 patients (1 man, 5 women, average age 44,6 years, all of them without any treatment), and C (control) group with 8 healthy participants (3 men, 5 women, average age 36,8 years). All patients took part in project “Healthy life with diabetes”. They were informed about experiment and related risks, and consents were obtained from all participants. The study protocol was approved by Bioethics Committee of the Medical University of Silesia -KNR/0022/KB1/82/II/15/16.

### Patient qualification

In this study we considered four clinical criteria for patients’ qualification: presence of type 2 diabetes based on level of HbA1c (normal range 4%-5,6%); BMI level (healthy weight:18,5–24,99 kg/m2; overweight: 25 ≤ kg/m2); lipid parameters disorders (healthy levels: LDL ≤ 100 mg/dL; HDL ≥ 60 mg/dL; TG ≤ 150 mg/dL) and insulin resistance based on HOMA-IR (optimal range ≤ 1.9).

The metabolic qualification was performed by assessment of carbohydrate and lipid disturbances in the blood serum: glucose level and HbA1c (glycated haemoglobin), the level of insulin resistance determined by HOMA - IR method and lipid profile (LDL cholesterol, HDL cholesterol, TG – triglycerides). Additional measurements included anthropometric parameters with body mass index (BMI), body composition using bioimpedance analysis and the assessment of fatty liver using ultrasound imaging methods. Characteristic**s** of metabolic parameters has been shown in Table 1. Other parameters relating to general condition of the body: renal (creatinine) and liver indicators (AlAt, AspAT), thyroid hormones (TSH), blood morphology, and inflammation marker (CRP) were assessed and were in normal level (data not shown).

**Table 1:** Characteristic**s** of metabolic parameters

Parameters	Unit	T2D group (mean/median)	IR group (mean/median)	Control group (mean/median)
Age	years	45.3/44	44.6/42	36.8/35.5
Sex	–	3♂ 6♀	1♂ 5♀	3♂ 5♀
BMI	kg/m2	38.1/39	24/25	24/24
Visceral index (bioimpedance)	–	13.6/11.5	6.3/5.5	4.3/4
Fasting plasma glucose	mg/dl	145/130	79/80	77/78
HbA1c	%	7.2/7.2	5.2/5.3	5.2/5.3
HOMA-IR	–	11.5/9.6	2.3/2.6	1.3/1.3
HDL (high-density lipoprotein)	mg/dl	40/41	69/73	64/63
LDL (low-density lipoprotein)	mg/dl	94/91	125/130	111/111
TG (triglycerides)	mg/dl	183/152	115/102	83/81
Liver fat in ultrasound imaging	–	present	absent/present	absent
Serum Insulin	μU/ml	33/32.1	11.6/13.7	6.9/6.7

Patients from IR group and C patients did not suffer from type 2 diabetes and they did not use any medicines in comparison to T2D group. However, some patients were characterized by BMI level over 25 kg/m^2^ and dyslipidemia (higher than healthy level of low-density lipoproteins- LDL and triglycerides). Liver fat in ultrasound imaging was also observed. Moreover, some of examined healthy volunteers had level above 1.9 of HOMA-IR.

Based on these findings, potentially healthy volunteers were classified into the IR group because they fulfill criteria of insulin resistance as a prediabetes state when blood glucose level is higher than normal but not high enough to be diagnosed as diabetes.

### Isolation of adipose derived stem cells

Human mesenchymal stem cells were isolated from selected patients as was described (Bunnell et al. 2008; Cheng et al. 2011; Francis et al. 2010). Adipose tissue samples were washed with phosphate-buffered saline (PBS) containing antibiotics: penicillin/streptomycin (Lonza, Switzerland) and amphotericin B (Lonza, Switzerland). Then, adipose samples were minced and incubated in collagenase type I (Lonza, Switzerland) for 2 h at 37 °C with shaking. The collagenase digestion reaction was stopped by adding DMEM medium with 10% fetal bovine serum. Stromal vascular fraction (SVF) was obtained by centrifuging at 1200 RPM for 5 min. The upper oil fraction was discarded. The pellet was transferred into culture dishes, resuspended and incubated overnight for selection of adherent cells in DMEM medium (Dulbecco’s Modified Eagle Medium, Lonza, Switzerland). Adherent cells were passaged when they reached 70% confluence (Bunnell et al. 2008; Cheng et al. 2011).

### Cell culture conditions

Normal human adipose derived stem cells (ADSCs) were routinely maintained in DMEM medium (Skubis et al. 2017; Sikora et al. 2019), supplemented with fetal bovine serum (FBS, EuroClone, Italy), amphotericin B and a penicillin-streptomycin mixture at 37 °C in a 5% CO_2_ incubator (Direct Heat CO_2_; Thermo Fisher Scientific, USA).

The culture medium was changed at intervals of 3 days. The experiment was performed on cells in the logarithmic phase of growth under condition of ≥98% viability assessed by trypan blue exclusion. Cells were assessed using the Olympus IX81 microscope (Olympus, Shinjuku, Tokyo, Japan) and DP70 camera (Olympus, Shinjuku, Tokyo, Japan) was used to photographic documentation. The ADSCs used for the experiment were at 2^nd^ passage, cultured in 6-well, 12-well and 96-well plates.

### Cell proliferation assays

Adipose derived stem cells were plated at density of 3 × 10^3^ per well in 96-well plates and incubated for 72 h. After that WST-1 (Roche, Switzerland) and sulforodamine B (SRB, Sigma-Aldrich, USA) assays were done.

For WST-1 absorbance of dye was measured at a wavelength of 450 nm and for SRB assay absorbance of dye was measured at a wavelength of 590 nm in 96-well plate (Thermo Fisher Scientific, USA) using Microplate Reader Perkin Elmer Wallac Victor 2 (Perkin Elmer, USA). All the tests were performed in septuplicate.

### Flow cytometry analysis

Human Mesenchymal Stem Cell Verification Flow Kit (FMC020, R&D Systems, USA) was used for mesenchymal stem cells identification. Kit includes all of the antibodies required for assessing MSC marker expression according to the International Society Cell Therapy’s definition of human MSCs and the appropriate isotype positive and negative controls. Kit contains conjugated antibodies for positive markers (CD73-CFS, CD90-APC, CD105-PerCP) and negative markers (CD45-PE, CD34-PE, CD11b-PE, CD79A-PE, HLA-DR-PE). Cell fluorescence was measured immediately after staining (FACS Aria 2; Becton Dickinson), and data were analyzed using software (BD FACSDiva Software, Becton Dickinson). The results are expressed as counts per 10,000 events. The frequency of positive cells was measured as the percentile of gated cells in fluorescent channels with activities of the corresponding isotype controls.

### Osteoblast differentiation

For these experiments, 20.000 cells per well were plated into 12-well cell culture plates. Second passage cells were used in the following studies. Cells were plated and grown until 75% confluent. Subsequently, the medium was replaced with fresh DMEM and 10% FBS, 50 μM L-ascorbic acid 2-phosphate, 10^− 7^ M dexamethasone and 10 mM β-glycerophosphate (Sigma-Aldrich, USA). Cells were cultured for 21 days. After that cells were collected and stored in − 20 °C until next analysis and Alizarin Red S (Sigma-Aldrich, St Louis, MO, USA) staining was made. Sample of every patient were cultured in triplicate.

### Alizarin red staining

Alizarin red staining is used to evaluate calcium deposits by cultured cells. Alizarin red at concentration of 40 mM was prepared in dH_2_O and the pH was adjusted to 4.1 using 10% ammonium hydroxide. For quantification of staining, 10% acetic acid (Sigma-Aldrich, USA) was added to each well for dissolving calcium deposits. The supernatants were read at 405 nm in 96-well plate using Microplate Reader Perkin Elmer Wallac Victor 2.

### Quantitative real-time polymerase chain reaction assay

Total RNA was extracted from cells using a TRIzol reagent (Invitrogen, USA). RNA extracts were treated with DNase I (MBI Fermentas, Lithuania) according to the manufacturer’s instructions. RNA concentration was determined using a GeneQuant II RNA/DNA spectrophotometer (Pharmacia Biotech, UK).

Expression assessment of *MKI67* and *phospho-histone H3 (pH 3), ALP, RUNX2*, *BGLAP, SPP1* was carried out using a real time RT-qPCR technique with SYBR Green chemistry (SYBR Green Quantitect RT-PCR Kit, Qiagen, Germany) and Opticon™ DNA Engine Continuous Fluorescence detector (MJ Research, USA) as described previously (Strzalka et al. 2008). All samples were tested in triplicate. β-actin was included as an endogenous positive control (housekeeping gene) of amplification and integrity of RNA extracts. Oligonucleotide primers (*MKI67, ALP, RUNX2, BGLAP, SPP1*) were obtain in Sigma-Aldrich company (Sigma-Aldrich, USA). The primers for amplification of *pH 3* mRNA were designed using Primer Express 1.0, ABI PRISM (Applied Biosystems, USA) (Orchel et al. 2004). Each reaction was completed using melting curve analysis to confirm the specificity of amplification and the absence of primer dimers.

### Statistical analysis

Statistical analyses were performed using Statistica 13.0 software. Values were expressed as median value (Me) with the 25^th^ and 75^th^ quartiles, and minimum and maximum for non-normally distributed data and for normally distributed variables are presented as mean and standard deviation. Different groups were compared using Kruskal-Wallis test for non-normally distributed data and ANOVA with post hoc Tuckey for normally distributed. The level of significance was set at *p* < 0.05 for all statistical tests.

## Results

### Analysis of viability in the ADSC – WST-1 and SRB assays

Cell viability was determined using the WST-1 proliferation assay, which is based on mitochondrial activity. Results obtained from the assay showed that the proliferation of cells from patients from the IR group was the highest (one-way ANOVA, post hoc Tukey, *p* < 0,05). We observed a statistically significant increase in viability in cells from IR patients as compared to control cells (*p* = 0.0001). The proliferation of cells from the T2D group was significantly lower than for the control cells (*p* = 0.0083). Moreover, a statistically significant decrease in viability was observed in cells from T2D patients compared to the IR group (*p* = 0.0001).

**Fig. 1:**
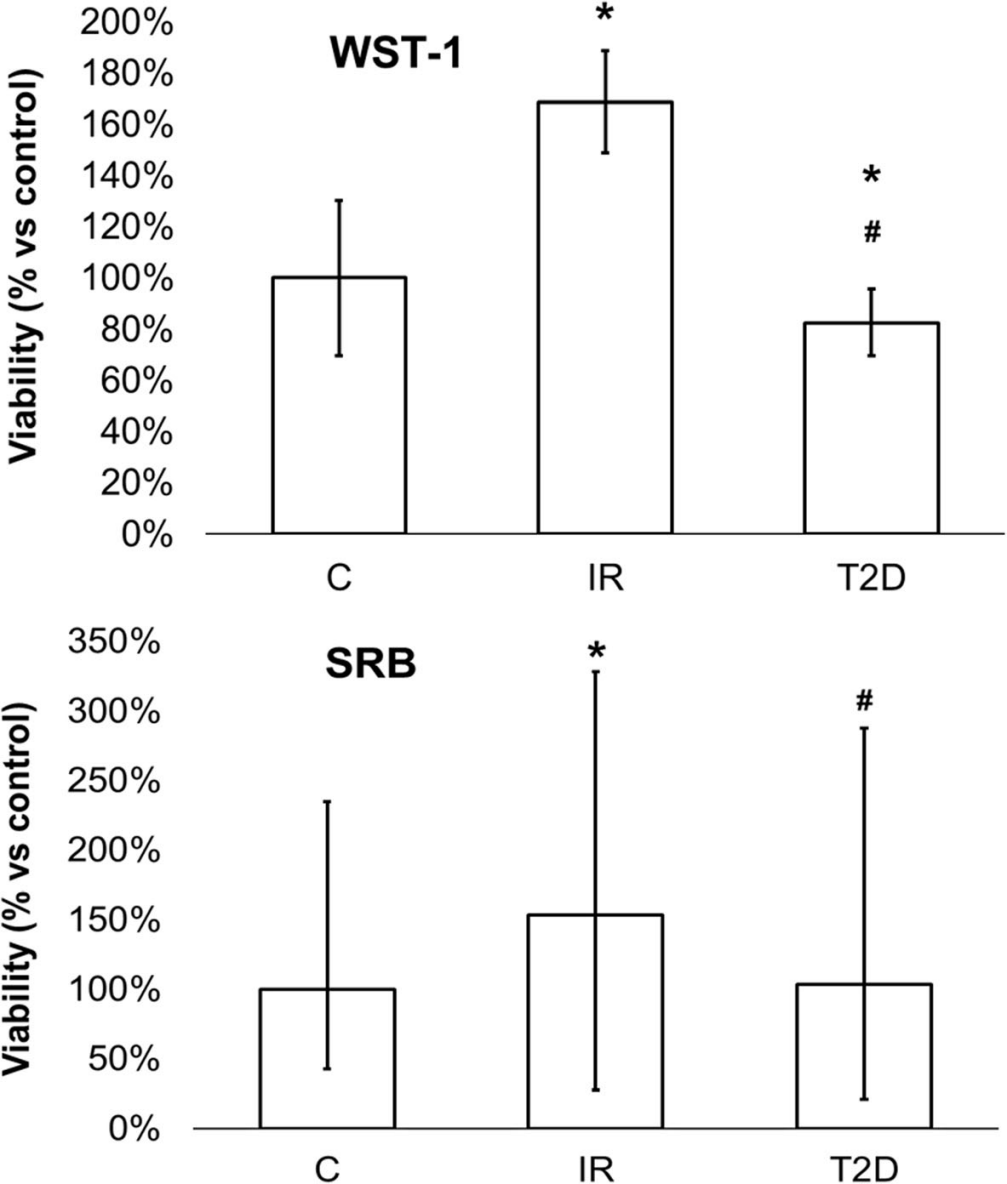
Cells viability based on the measurement of mitochondrial oxidative (WST-1 assay) in cells and total protein content (SRB assay) from patients with insulin-resistance (IR), type 2 diabetes (T2D) compared to control cells (C) from healthy people. The bars represent the means ± standard deviation (SD), ANOVA with the Tukey post hoc test (WST-1 assay) and the (Me) with the 25^th^ and 75^th^ quartiles and the minimum and maximum; the Kruskal Wallis test with post hoc (SRB assay), *p < 0.05 vs. C, #*p* < 0.05 vs. IR

The cell viability was also assessed by measuring the total protein content in cells (SRB test) in all examined groups (Kruskal-Wallis test, *p* < 0.05). Cells from IR patients showed higher viability compared to the control cells (*p* < 0.0001) and a statistically significant higher viability than T2D cells (*p* = 0.0036). Moreover, there was no difference in cells from T2D patients compared to control cells (Fig. 1).

### Analysis of *MKI67* and *pH 3*

**Fig. 2:**
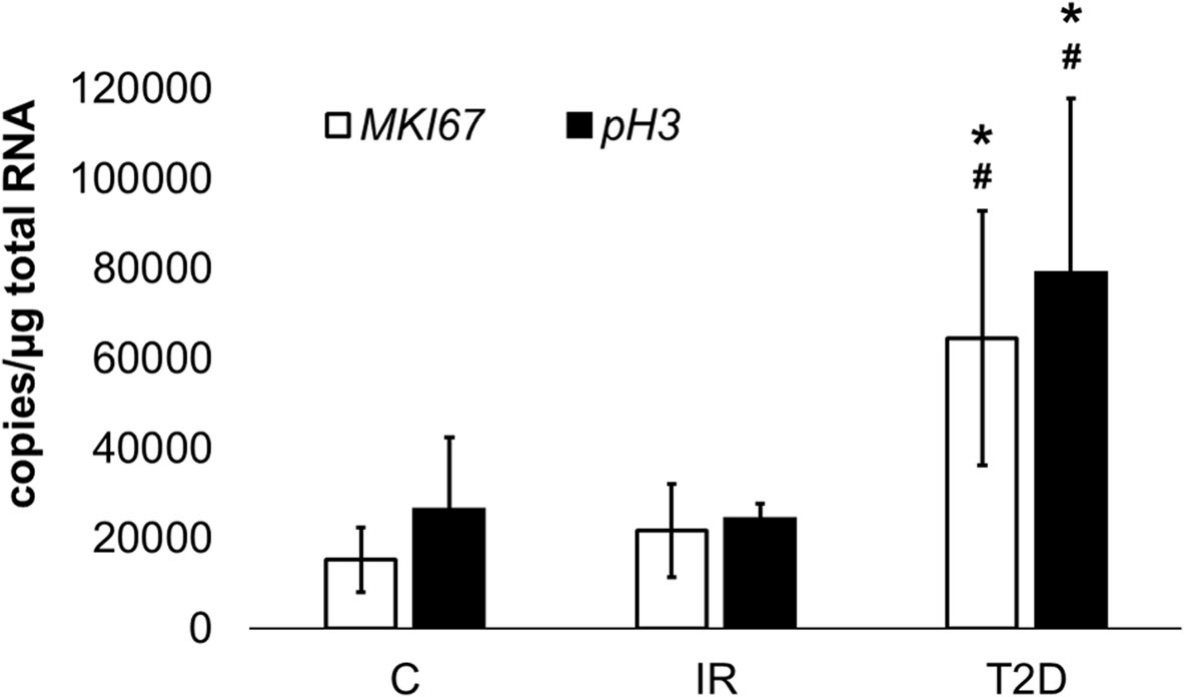
The mRNA levels of *MKI67*and *pH 3* in the ADSC from patients with insulin-resistance (IR), diabetes mellitus type 2 (T2D) compared to control cells (C) from healthy people. The bars represent the means ± standard deviation (SD) of the copy numbers per 1 μg of total RNA; ANOVA with the Tukey post hoc test *p < 0.05 vs. C, #*p* < 0.05 vs. IR

An analysis of the mRNA levels of the *MKI67* and *pH 3* genes allowed for an estimation of the cell proliferation. The *MKI67* mRNA level was significantly higher in cells from T2D patients compared to the control (*p* = 0.0001) and IR cells (*p* = 0.0002). Similarly, the expression of *pH 3* was higher in cells from the T2D group compared to the control (*p* = 0.0001) and IR cells (*p* = 0.0002) (Fig. 2).

### Analysis of ADSC phenotype

**Fig. 3:**
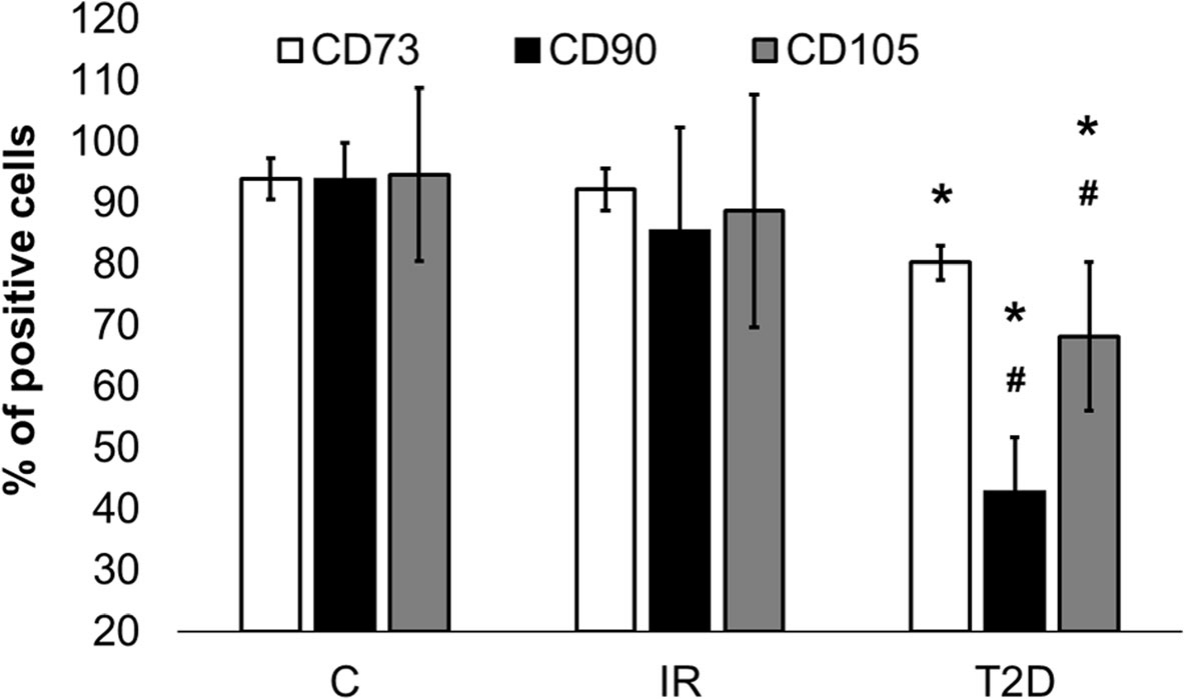
The percentage of positive cells for CD73, CD90 and CD105 markers in cells from patients with insulin-resistance (IR), type 2 diabetes (T2D) compared to control cells (C). The bars represent the means ± standard deviation (SD); ANOVA with the Tukey post hoc test *p < 0.05 vs. C, #*p* < 0.05 vs. IR

**Fig. 4:**
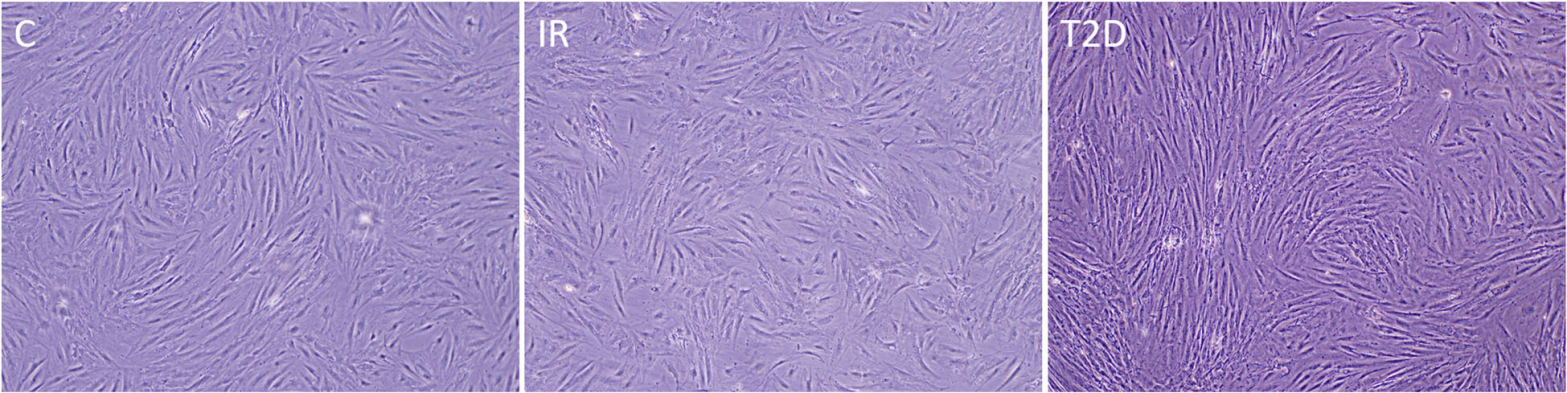
The morphology of ADSCs from patients with insulin-resistance (IR), type 2 diabetes (T2D) compared to control cells (C)

We performed a fluorescence-activated cell sorting (FACS) analysis, which showed the expression of cell surface CD73, CD90, and CD105-specific markers of MSCs to assess the phenotype of the examined cells. The percentage of marker-positive cells was different in the examined groups. CD73 expression was significantly higher in the T2D group compared to the control cells (*p* = 0.0129). The expression of CD90 was statistically significantly lower in T2D cells compared to control (*p* = 0.0002) and IR cells (*p* = 0.001). The analysis of CD105 also showed statistically lower expression in T2D cells compared to the control (*p* = 0.0007) and IR cells (*p* = 0.011). Moreover, the expression of CD105 was significantly lower in T2D cells compared to IR cells (*p* = 0.001) (Fig. 3). Also, morphology of cells from T2D, IR and C groups were analyzed and we did not observe any differences in shape of cells (Fig. 4).

### Analysis of the osteogenesis potential of ADSCs

**Fig. 5:**
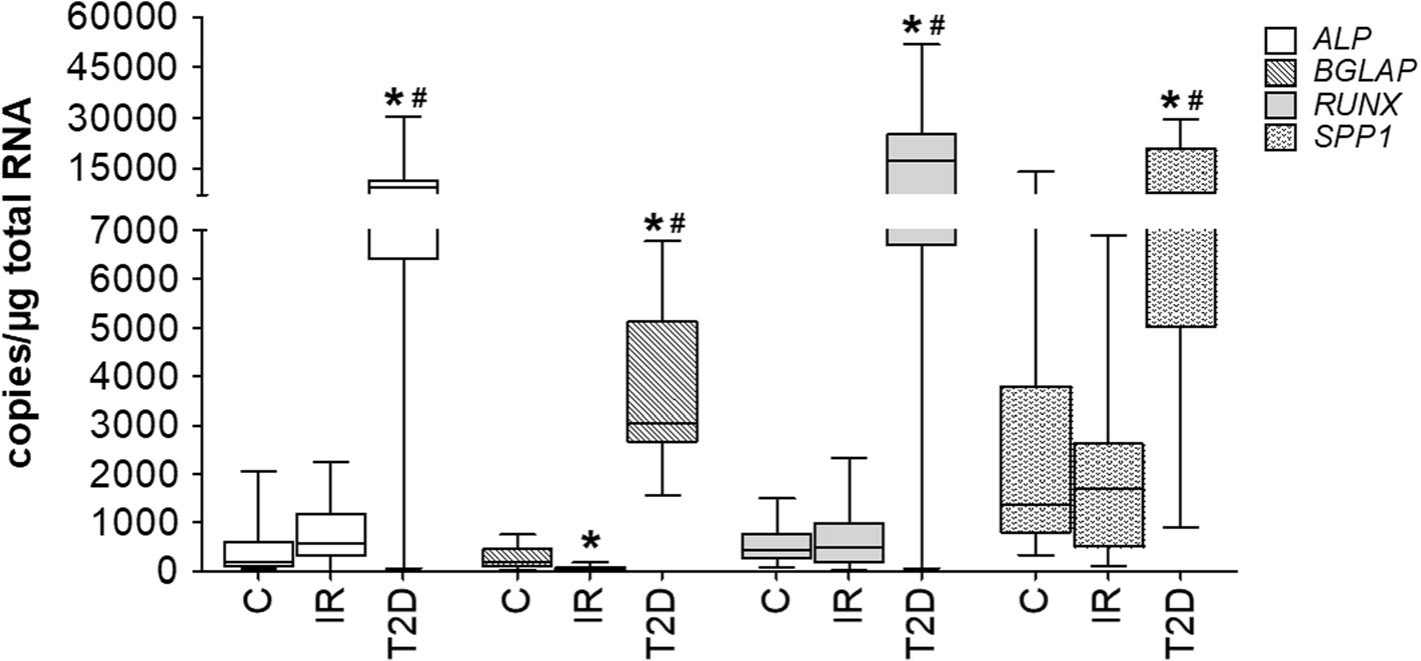
The mRNA levels of *BGLAP, ALP, SPP1, RUNX2* in the ADSC from patients with insulin-resistance (IR), type 2 diabetes (T2D) compared to control cells (C) from healthy people after osteoblast differentiation. The bars represent the (Me) with the 25^th^ and 75^th^ quartiles and the minimum and maximum of the copy numbers per 1 μg of total RNA; the Kruskal Wallis test with post hoc, *p < 0.05 vs. C, #*p* < 0.05 vs. IR

We observed a higher expression of *RUNX2* in cells in T2D group compared to the control cells (*p* = 0.0038) and cells from IR patients (*p* = 0.0104). Moreover, similar results were proved in the expression levels of *SPP1* and *ALP,* Expression was higher in T2D cells in comparison to the control (*SPP1*, *p* = 0.0001; *ALP*, *p* < 0.0001) and to cells from patients with insulin resistance (*SPP1*, *p* < 0.0001; *ALP*, *p* = 0.0263). Interestingly, the analysis showed that the expression of *BGLAP* was lower in the IR group versus the control (*p* = 0.0263). Additionally, the mRNA level of *BGLAP* was higher in T2D cells compared to the control (*p* < 0.0001). There was also a statistically significant higher expression of *BGLAP* in cells from the T2D group versus the IR group (*p* < 0.0001) (Fig. 5).

**Fig. 6:**
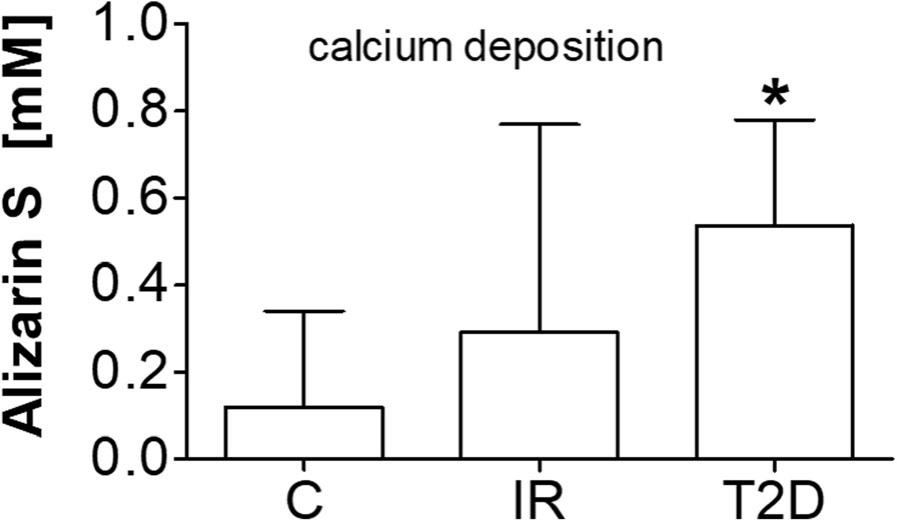
Alizarin red S concentration [mM] in examined groups; insulin-resistance (IR), type 2 diabetes (T2D) compared to control cells (C); ANOVA with the Tukey post hoc test *p < 0.05 vs. C

For osteogenesis, the presence of extracellular calcium was confirmed by Alizarin red staining. Calcium deposition was also analyzed after 21 days of culturing (Fig. 6). The calcium level was measured and expressed as mM of calcium. Samples were evaluated in triplicate. The analysis showed a statistically significant higher level of calcium in cells from T2D patients versus the control (*p* = 0.0001) and IR cells (*p* = 0.0028).

## Discussion

Type 2 diabetes is a metabolic disorder that begins with insulin resistance and is associated with a constantly high glucose level. The result of this abnormal insulin activity is a state of hyperglycemia, which contributes to a number of dysfunctions in body and tissues: the excessive production of reactive oxygen species in mitochondria (Jiao et al. 2015); the increase in protein oxidation, lipid peroxidation, and nucleic acid damage, all leading to cell damage (Jiao et al. 2015; Zabłocka and Janusz 2008); and chronic, difficult-to-soothe inflammation (Jiao et al. 2015). Additionally, nephropathy-impaired wound healing as well as irregularities in the bone and skeletal system have also been observed (Cheng et al. 2016).

Osteogenesis is a multistage process that is controlled in vivo by many molecular pathways. MSCs from adipose tissue can differentiate into mesodermal cells, such as osteoblasts, and they can be used in bone regeneration. Unfortunately, ADSCs differ between patients, especially due to a variety of diseases or drug use (Efimenko et al. 2015; Minteer et al. 2015), which can inhibit the efficacy of autologous cell therapy. Clinical studies have revealed that patients with T2D have more complications with bone healing (Dufrane 2017; Liang et al. 2014). Some studies have shown that diabetes alters the proliferation and activity of ADSCs (Marycz et al. 2016; Nawrocka et al. 2017; Serena et al. 2016).

In our research, we examined the proliferation of ADSCs from patients with IR and T2D. We analyzed the cell proliferation based on a mitochondrial activity assay and a total protein content measurement. It was confirmed by both assays that ADSCs from patients with IR demonstrated a higher proliferation compared to the control. This proliferation may result from IR, hormonal imbalances, and, consequently, constant higher levels of glucose (Ko et al. 2015; Lee et al. 2018). Moreover, only the WST-1 assay showed a decreased proliferation in T2D cells in comparison to IR cells. Our results also revealed the upregulation of *MKI67* and *pH 3,* molecular markers of proliferation (Orchel et al. 2004; Thompson et al. 2015), in cells from the T2D group compared to cells from the control and IR patients. It has been proven that medicines such metformin influences many molecular pathways (Hur and Lee 2015; Viollet et al. 2012), and it is likely that differences in the cytotoxicity assay results and the mRNA profiles may be related to the medicines taken by T2D patients, but further studies are required to confirm this observation.

However, the upregulation of *MKI67* and *pH 3* mRNA expression may be due to the fact that these cells were cultured in standard conditions in vitro with different glucose level in comparison to the conditions which were present in patients organisms. The reason of the upregulation of the proliferation markers gene expression may be associated with the potential regeneration of ADSC derived from diabetic patients. However, this effect on the metabolic level assessed by WST-1 and SRB assays could be yet undetectable, because the transcription and changes at the genome level precede significantly the phenotype changes where the metabolic activity and the protein production are included.

Our finding showed that cells from T2D patients have a lower expression of membrane proteins characteristic for stem cells: CD73, CD90, and CD105. The results did not show a cellular change for IR patients at the protein level.

Our study showed that the differentiation of ADSCs from T2D patients into osteoblasts was stronger than in cells from the control group or IR patients. Both type 1 and type 2 diabetes are associated with an increased risk of bone fractures. In the course of the disease, abnormal microarchitecture and quality of bone are seen, as well as irregularities in new bone formation (Räkel et al. 2008). Hyperglycemia resulting in decreased expression of the genes encoding for markers of osteoblast formation also leads to an increased expression of proinflammatory cytokines. This all contributes to a reduction in osteoblast activity and differentiation and induces their apoptosis. Another mechanism of hyperglycemia is the promotion of adipogenesis in MSCs using high glucose concentrations, which is connected to the inhibition of MSCs differentiation into osteoblasts by changing the differentiation towards that of adipocytes, instead (Yan and Li 2013). It was suggested that the high glucose levels inhibited the differentiation of osteoblasts and osteoblast precursors (Jiao et al. 2015; Li et al. 2010). Moreover, a high concentration of glucose leads to the previously mentioned formation of advanced glycation end products (AGEs) and reactive oxygen species (ROS) over-production. The final AGEs stimulate osteoclast formation and induce the osteoclastogenic process and inhibit the differentiation of osteoblasts by the decreased expression of alkaline phosphatase and collagen 1α1. There is also evidence that AGEs induce apoptosis of osteoblasts (Jiao et al. 2015). ROS stimulate the differentiation and survival of osteoclasts and promote apoptosis of osteoblasts. The long-term effect of oxidative stress is the reduction of bone mass (Jiao et al. 2015). Increased levels of TGF-β in the serum of people with diabetes leads to the reduced activity of alkaline phosphatase, which is responsible for providing the necessary phosphate in the matrix mineralization. The result is a decrease in matrix mineralization and abnormal bone formation (Ehnert et al. 2015). Inflammatory factors induce and maintain the regulation of these processes through the bone resorption by osteoclasts in the process of osteoclastogenesis. The inflammatory process in diabetes can reduce the number of osteoblasts via the induction of apoptosis. In diabetes, an increased expression of pro-apoptotic genes, including an increased Bax/Bcl-2 ratio, was reported, which corresponds with increasing of apoptosis (Jiao et al. 2015).

We observed a higher potential for osteogenesis based on the mRNA level of *BGLAP, SPP1, ALP,* and *SPP1* in patients with T2D. The same results were also observed in the Alizarin red staining for calcium deposition. We did not observe a significant change in IR patients in comparison to the control, suggesting that hyperglycemia is not a main differentiation factor.

Many studies suggest that metabolic diseases like diabetes inhibit the activity and differentiation ability of stem cells. Less is known about the influence of diabetes pharmacotherapy on stem cell function, especially in human models. Most studies have focused on animal models, though there are many factors that change the activity of cells in humans. Apart from diabetes treatment, other factors might have influenced these results, including comorbidities, age, and length of disease. Such factors cannot be assessed in animal models.

## Conclusion

We have concluded that type 2 diabetes changes the activity of stem cells, and insulin resistance influences on the proliferation of ADSCs.

## Data Availability

Not applicable.

## Abbreviations

ADSCs: Adipose-derived stem cells
AGEs: Advanced glycation end products
AlAt: Alanine aminotransferase
*ALP*: Alkaline phosphatase
ANOVA: Analysis of variance
APC: Allophycocyanin
AspAT: Aspartate aminotransferase
BMI: Body mass index
*BGLAP*: Osteocalcin
CD105: Endoglin
CD73: Cluster of differentiation 73/ecto-5′-nucleotidase
CD90: Cluster differentiation 90/Thy-1 antigen
CD45: Protein tyrosine phosphatase/ receptor type C
CD34: Hematopoietic Progenitor Cell Antigen CD34
CD11b: Integrin subunit alpha M/ITGAM
CD79A: B-cell antigen receptor complex-associated protein alpha chain
CFS: Carboxyfluorescein
CRP: C-reactive protein
dH2O: Distilled water
DMEM: Dulbecco’s Modified Eagle Medium
DNA: Deoxyribonucleic acid
FACS: Fluorescence-activated cell sorting
FBS: Fetal bovine serum
HbA1c: Glycated haemoglobin
HDL: High-density lipoprotein
HLA-DR: Human Leukocyte Antigen – DR isotype
HOMA - IR: Homeostatic model assessment for insulin resistance
IR: Insulin resistance
LDL: Low-density lipoprotein
mE: Median
*MKI67*: Marker of proliferation Ki-67
mRNA: Messenger ribonucleic acid
MSC: Mesenchymal stem cells
PBS: Phosphate buffered saline
PE: Phycoerythrin
PerCP: Peridinin-chlorophyll-protein
*pH 3*: Phospho histone h3
RNA: Ribonucleic acid
ROS: Reactive oxygen species
RTq PCR: Quantitative real-time polymerase chain reaction
*RUNX2*: Runt-related transcription factor 2
*SPP1*: Osteopontin
SRB: Sulforhodamine B
SVF: Stromal vascular fraction
T1D: Type 1 diabetes
T2D: Type 2 diabetes
TG: Triglyceride
TGF-β: Tansforming growth factor beta
TSH: Thyrotropic hormone
WST-1: (4-[3-(4-Iodophenyl)-2-(4-nitrophenyl)-2H-5-tetrazolio]-1,3-benzene disulfonate)
